# Visualization support for remote collaborative aneurysm treatment planning

**DOI:** 10.1007/s11548-025-03508-9

**Published:** 2025-09-08

**Authors:** Rebecca Preßler, Monique Meuschke, Bernhard Preim, Kai Lawonn

**Affiliations:** 1 Institute of Computer Science, Friedrich-Schiller-Universität, Fürstengraben 1, 07743 Jena, Thuringia Germany; 2 Department of Simulation and Graphics, Otto-von-Guericke-Universität, Universitätsplatz 2, 39106 Magdeburg, Saxony-Anhalt Germany

**Keywords:** Aneurysm, Collaborative application, Visualization, Computer graphics

## Abstract

****Purpose**:**

Cerebral aneurysms are blood-filled bulges that form at weak points in blood vessel walls, and their rupture can lead to life-threatening consequences. Given the high risk associated with these aneurysms, thorough examination and analysis are essential for determining appropriate treatment. While existing tools such as Aneulysis and its web-based counterpart WebAneulysis provide interactive means for analyzing simulated aneurysm data, they lack support for collaborative analysis, which is crucial for enhancing interpretation and improving treatment decisions in medical team meetings.

****Methods**:**

To address this limitation, we introduce WebCoAneulysis, a novel collaborative tool for aneurysm data analysis. WebCoAneulysis builds upon the established visualization techniques of WebAneulysis while incorporating innovative collaborative features to facilitate joint analysis and discussion among medical professionals. The tool was evaluated by three physicians and two visualization experts, who assessed its usability, functionality, and effectiveness in supporting collaborative decision-making.

****Results**:**

The evaluation results were overwhelmingly positive. The physicians particularly appreciated the tool’s ability to provide a clear overview of aneurysm data while maintaining ease of use despite its complex functionality. Although minor suggestions for improvement were made, the overall feedback highlighted the benefits of WebCoAneulysis in improving collaborative analysis and treatment planning.

****Conclusion**:**

WebCoAneulysis enhances aneurysm data analysis by enabling real-time collaboration among medical professionals, thereby supporting more informed treatment decisions. Beyond its primary application in risk analysis and treatment planning, the tool also has potential benefits for patient education and the training of new doctors, making it a valuable addition to the field of medical visualization and decision support systems.

**Supplementary Information:**

The online version contains supplementary material available at 10.1007/s11548-025-03508-9.

## Introduction

Cerebral aneurysms occur in approximately 3–5 % of the general population [1]. The exact incidence is difficult to determine, as most aneurysms are discovered incidentally, with unruptured aneurysms typically causing no symptoms [2]. When an aneurysm ruptures, blood enters the subarachnoid space, a condition that can be fatal for the patient. Despite advancements in treatment, the mortality rate remains around 40 % [3].

In clinical practice, the shape, size, and location of an aneurysm typically determine the course of treatment. However, these criteria are not reliable for predicting the risk of rupture. Rupture seems to depend on the interplay between blood flow and aneurysm wall characteristics. To better understand these relationships, blood flow simulations are performed. These simulations are based on 3D vascular models derived from medical imaging techniques like CTA and MRA, resulting in complex, time-dependent 3D data.

Previous research has focused on developing reliable workflows for creating patient-specific vascular models [4–6] as well as visualization tools for the interactive exploration of simulated aneurysm data, such as Aneulysis [7] and WebAneulysis [8]. These tools are designed to assist physicians in making informed decisions regarding the most appropriate course of treatment. However, they lack collaborative analysis capabilities, which are crucial for improving data interpretation and treatment decisions through expert collaboration.

With this in mind, our goal is to enhance the collaborative features of WebAneulysis [8], enabling us to introduce a collaborative, multi-visualization tool for aneurysm analysis. The new collaborative functionality enables physicians to engage in meaningful discussions with colleagues and other medical experts while accessing cases remotely.

After conducting informal interviews with two physicians to understand their needs and preferences for a collaborative application, as well as drawing insights from similar studies [8–10], new features like a video chat and view sharing were added. The updated version of WebAneulysis, WebCoAneulysis, was subsequently presented to a group of physicians and computer scientists for evaluation. The results, along with a detailed overview of the new workflow and features, are outlined in the following sections.

## Related work

This section gives a short overview of existing visualization and communication tools and their purposes.

### Visualization of aneurysm data

There are numerous visualization approaches and analytical tools for aneurysms: *AngioLab* was developed as an extension of the open-source biomedical image computing and simulation framework *GIMIAS* [11]. *AngioLab* offers an advanced morphological analysis and endovascular treatment planning [12]. *AView* was developed to facilitate treatment decisions and planning in or near the procedure room. This clinical tool integrates the computation of hemodynamics and morphology, as well as risk and data management [13]. *Ankyras* facilitates the sizing of flow-diverting stents [14]. The software is capable of creating anatomical models from 3D MRA images, computing and measuring morphological parameters, and creating and analyzing flow-diverting stents tailored to the specific anatomy of the patient. Arrieta Llorente designed a web application to support the analysis of cerebrovascular image data [15]. Three graphs were created that help analyze the temporal blood flow and skeleton of the vascular system to identify vascular abnormalities and malformations in the brain.

The aforementioned applications are distinguished by the set of tools they offer. However, none of them provides the functionality to support a multiuser workflow.

In 2020, Meuschke et al. developed and released a comprehensive desktop application, entitled Aneulysis [7]. This software provides a visual exploration of aneurysm data sets. It integrates three key elements of aneurysm rupture risk and treatment research: the analysis of morphological data, the exploration of flow patterns, and the simultaneous investigation of both. Thus, Aneulysis comprises a variety of analytical tools arranged in five modules. For example, it is possible to compute and extract morphological descriptors of aneurysms (like the diameter or height of the bulge) or to study vessel wall thickness and deformation concerning blood flow. Aneulysis received promising feedback, yet it became evident that sharing the analytical results was a challenging endeavor.

To address this issue, the *WADE—Web-based Aneurysm Data Exploration* module was incorporated into the Aneulysis software. The module provides a web interface that displays a dataset exported from the locally installed Aneulysis tool and its corresponding analysis results. As a result of the uploaded dataset being accessible via a web server, physicians, patients, and students who lack comprehensive database access can now view the analytical results [16]. In order to address the limitations of local device dependency, like installation restrictions, Aneulysis has been reworked as a web application, implemented with JavaScript [8] WebAneulysis operates in conjunction with the *THREE.js* library and is accessible via an internet browser. It incorporates three distinct interactions and analytical features, specifically:

- Landmark addition to share interesting exploration findings
- Stent placement
- Morphological parameter calculation

The calculation of morphological parameters is a function of the desktop application of Aneulysis [7] The landmark addition and stent placement are entirely novel for the web-based version of this application.

An alternative visualization method, developed as an enhancement to the *WADE* module, is the virtual DSA [17]. This approach uses a specialized shader to convert the vivid, CFD-calculated blood flow streamlines into a representation that more closely mirrors a traditional DSA visualization. For experienced physicians, who may be more accustomed to conventional visualizations, the colorful streamline-based imagery can sometimes be difficult to interpret. The virtual DSA provides users with the flexibility to adjust the visualization, presenting it in a format that aligns more closely with their familiarity and expertise.

### Web communication tools

The importance of long-distance communication tools has increased significantly in recent times, particularly in the context of the Coronavirus pandemic and the associated lockdowns. This has resulted in the emergence of novel challenges with regard to the optimal utilization and design of communication tools.

The multitude of available applications, which vary in terms of setup, price, usability, and functionality [18], has led to a need for careful consideration of their appropriate use in different contexts. The advantages and disadvantages of the various tools are explored [19, 20].

In the creation of a communication tool for collaborative purposes, it is of particular importance that the feature is straightforward to use and facilitates the collaborative work process. Primarily, the objective is to facilitate the *remote synchronous* observation of patients by physicians. But the provided visualization can also be utilized in a colocated situation when both physicians are situated in the same location.

To circumvent restrictions related to the installation of software on clinical computers, our design does not involve the installation of local software. To ascertain the configurations preferred by users in video conferences, Balogova et al. [10] conducted a study with 115 participants. In a meeting with a limited number of participants and a need for collaboration, the researchers found that users preferred the grid view, which allowed them to view the videos of all logged-in users. Moreover, the majority of participants expressed a preference for viewing their video to assess their appearance. Given that physicians frequently collaborate with colleagues and experts, although often in small groups or even as a pair, these findings are noteworthy for our design of the feature in question.

Data protection regulations are another issue related to web applications. According to Article 9 of the general data protection regulation (GDPR), patient data is considered particularly worthy of protection, and the processing of this data is subject to specific requirements, which are discussed in more detail in Article 32 of the GDPR. In the initial phase of developing a prototype that uses anonymized test data, these requirements are to be set aside so that development efforts may be concentrated on new functions for the WebCoAneulysis program. In the event that the concept demonstrates success in a preliminary trial, it would be feasible to subsequently implement and host the tool following the GDPR.

## Requirement analysis

In order to ascertain the essential requirements for a collaborative platform dedicated to aneurysm data analysis and treatment planning, two neuroradiologists were interviewed, who both have over ten years of professional experience and routinely analyze and treat aneurysm patients. The aim was to pinpoint the specific needs of these medical professionals and integrate them into the platform’s design. Each physician was interviewed individually and asked whether they currently have access to analytical or visualization tools, whether they actively use these tools, and if they could envision any additional features that would enhance their daily work.

Both neuroradiologists currently lack access to advanced visualization tools, such as Aneulysis, for risk assessment and treatment planning of aneurysms. Instead, their evaluations rely solely on contrast-enhanced imaging data. Nevertheless, both doctors were convinced of the benefits of advanced visualizations and even proposed additional enhancements to the tool. One neuroradiologist suggested extending the landmark feature (a tool that allows small torus-shaped marks to be placed on the displayed model) with more diverse geometries to improve the visibility of different marking points. Also, the utility of a commentary function was discussed, whereby physicians could annotate landmarks of other users. Furthermore, a proposal was made regarding the stent function, namely the addition of a diameter configuration, which would allow for the alteration of the placed stent after its addition to the model.

Moreover, they found the visualization of the aneurysm model’s multi-parameter data and the associated interaction features particularly valuable. They would welcome the ability to interact with the dataset and also suggest the implementation of a screen-sharing tool. Thus, the user should be provided with the option of switching between different modes, either to display their interactions or to view those of a different user. When the user views the interactions of a different user, the user’s interactions should be restricted to a minimum to avoid disturbing the sharing. The relevant modes should be easily accessible at any time.

Both physicians noted that having a single tool to view different types of data and access various features would be highly beneficial. They observed that the current process of switching between separate applications for each data type, like the simulated blood flow visualizations and created vessel wall models, is time-consuming, especially during calls or meetings that also require an additional communication platform. They emphasized the need for an integrated platform that combines all essential functions—data viewing, interaction, and communication—into one application.

The second neuroradiologist highlighted a valuable application area for such a platform: patient education. In his daily practice, he interacts extensively with patients, often needing to explain treatment options and illustrate procedures. This process is time-consuming and requires detailed documentation. He expressed interest in using a tool like Aneulysis to provide enhanced visual support during patient consultations and suggested an export feature for documentation, which would allow him to focus more on patient dialogue.

Finally, both physicians mentioned that they frequently discuss treatments with colleagues and also review past cases to inform treatment decisions. They, therefore, proposed a solution whereby multiple datasets may be opened and interacted with in a single session without the loss of progress on any of these datasets.

In conclusion, the physicians expressed satisfaction with the concept of the application and provided constructive suggestions for its future development. Based on the interviews, we derived the following requirements (R1–R7) for WebCoAneulysis:

***Visualization-related requirements***

1. Multiple geometries for landmarks
2. Stent diameter configuration
3. Model and dataset selection

***Communication-related requirements***

R4 Multiple user support
R5 Landmark commentary function
R6 Integrated communication tools
R7 Different work modes for screen sharing

## Collaborative aneurysm analysis

**Fig. 1:**
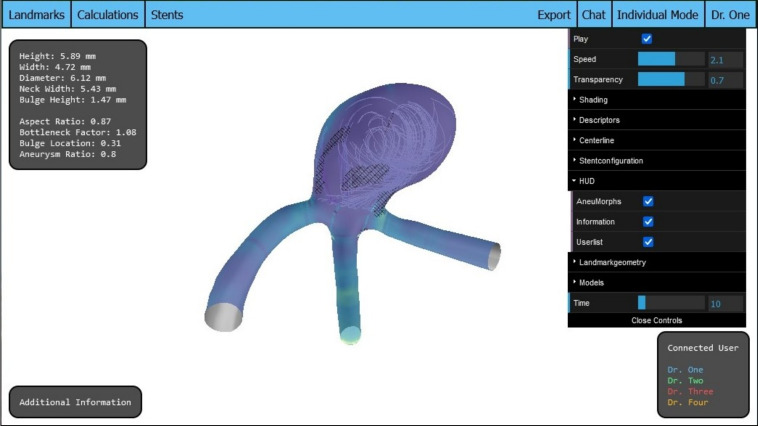
Overview of the application’s interface, which features a header for the primary functionalities and boxes for the heads-up display (HUD) elements. These boxes encompass the morphological parameters, supplementary data, and the user list. In the upper right corner, the control panel offers additional settings

WebAneulysis serves as the basis for WebCoAneulysis. Core design elements of WebAneulysis are preserved, such as the central display of the aneurysm model and the text-based information boxes located at the screen’s corners, see Fig. 1. These components provide a sound basis upon which collaborative features are integrated, enhancing the tool’s utility for team-based analysis.

The header, which lists accessible features for landmark and stent placing as well as the morphological parameter calculation, is located at the top of the screen, while a control menu for further interaction and analyzing tools is set on the right side. A comprehensive description of this can be found in Preßler et al. [8]. WebCoAneulysis is written in *HTML* with *CSS* and *JavaScript* and utilizes the *THREE.js* library. In order to accommodate forthcoming extensions, the *Socket.io* and *PeerJS* libraries will also be required. The *Socket.io* library facilitates bidirectional communication between a server and a client via sockets, which are objects from the operating system used for communication. The *PeerJS* library enables the establishment of a peer-to-peer connection using the browser’s WebRTC implementation, requiring only a single ID for the connection. Both media and data streams can be established.

### Multiple user support

To meet R4, the initial step was to establish a connection between the client and server for subsequent collaborative processes. On the client side, a user login is required, and on the server side, all logged-in users must be accepted, and a communication channel between them must be established.

In essence, sockets operate using an event-driven architecture. Typically, the methods socket.on and socket.emit, along with a string representing the event name, are fundamental for enabling communication. Based on this structure, various events must be defined to track and log user connections and disconnections, maintain and update a list of all logged-in users, and share this information with all active clients. This ensures visibility into who is currently online. Additionally, any changes made by a user, such as the addition of landmarks or flow-diverting stents, must be reflected in the views of all other logged-in clients. This guarantees that every client has access to the most up-to-date information.

In order to facilitate the monitoring of alterations made by disparate clients, colors are used. Upon successful authentication, a distinct color is allocated to each user. The name displayed on the user list, the landmarks and stents that have been placed, as well as the header of the view are colored according to the unique user color. Consequently, at first glance, users can discern which changes have been made by which user. The color assignment is conducted on the server side to guarantee that each color is exclusively assigned once.

The fundamental overview of the application, with all information boxes activated and four users logged in, is illustrated in Fig. 1.

### Revision of existing features

Starting with the landmark feature and the addition of new geometries (R1), a drop-down selection element has been added to the controls menu on the right side, allowing users to choose between three shapes for the landmark: the previously implemented torus, a cube, or a sphere. These geometries were chosen because they are easy to distinguish from each other. Currently, only three geometries are available so as not to overwhelm the test users with a large selection. If it becomes clear that more geometries are needed, this feature could easily be extended.

**Fig. 2:**
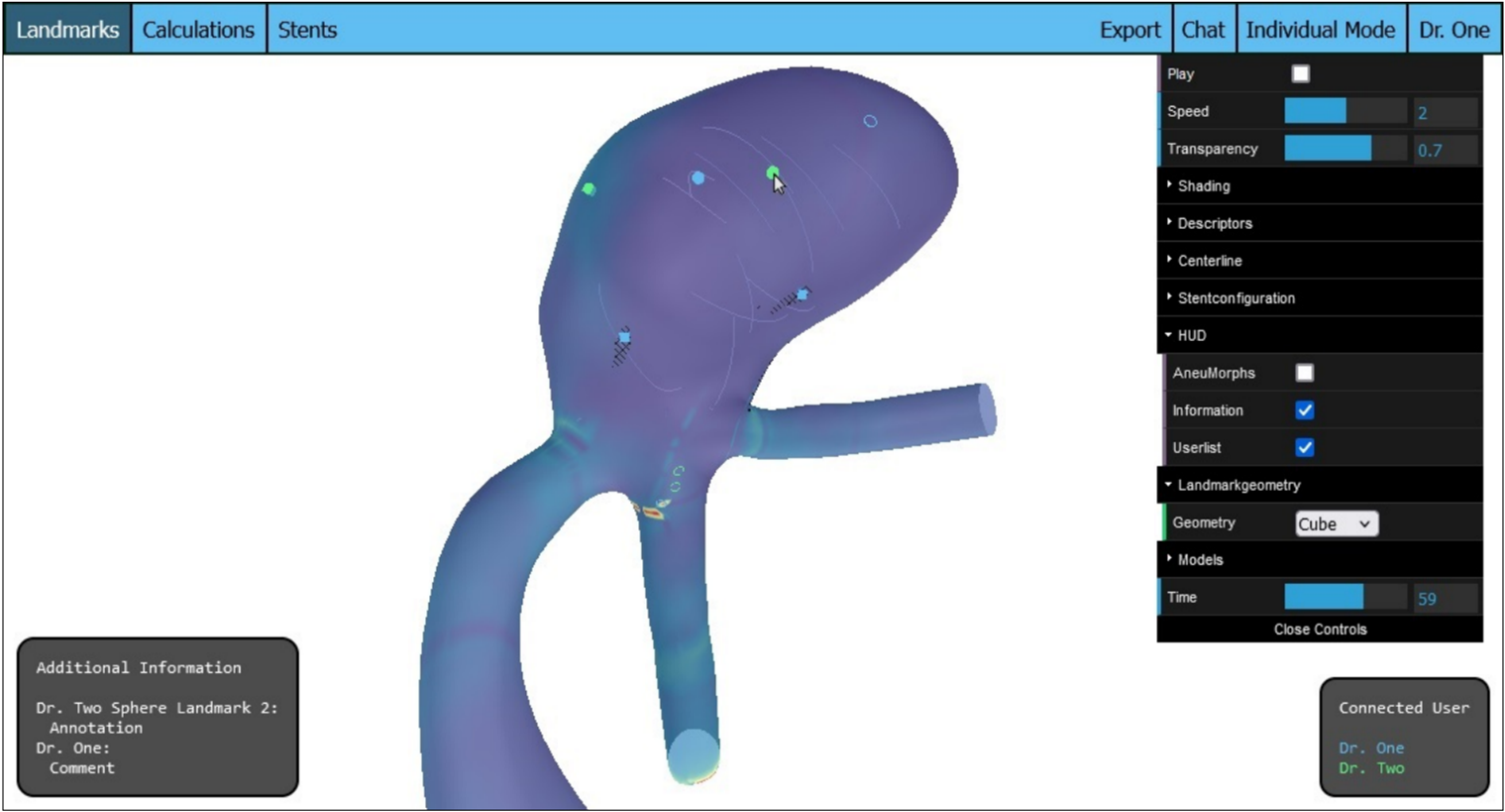
Multiple shaped, placed landmarks from different users. The annotation and comment of a hovered landmark is displayed

The new commentary function (R5) operates similarly to the landmark annotation feature. When the user hovers over a placed landmark, they can click to leave a brief note. This hovering action is detected through ray casting, where a ray is projected from the cursor through the scene, capturing all intersected objects in an array. If the first entry in this array is an existing landmark, the annotation function is triggered. Extending this ray-casting verification, the commentary function will activate if the hovered object is a landmark belonging to another user, prompting the user to enter a note. Similar to the annotations displayed in the information text box, the commentary can be viewed by hovering over the respective landmark. Figure 2 illustrates the refined landmark feature.

**Fig. 3:**
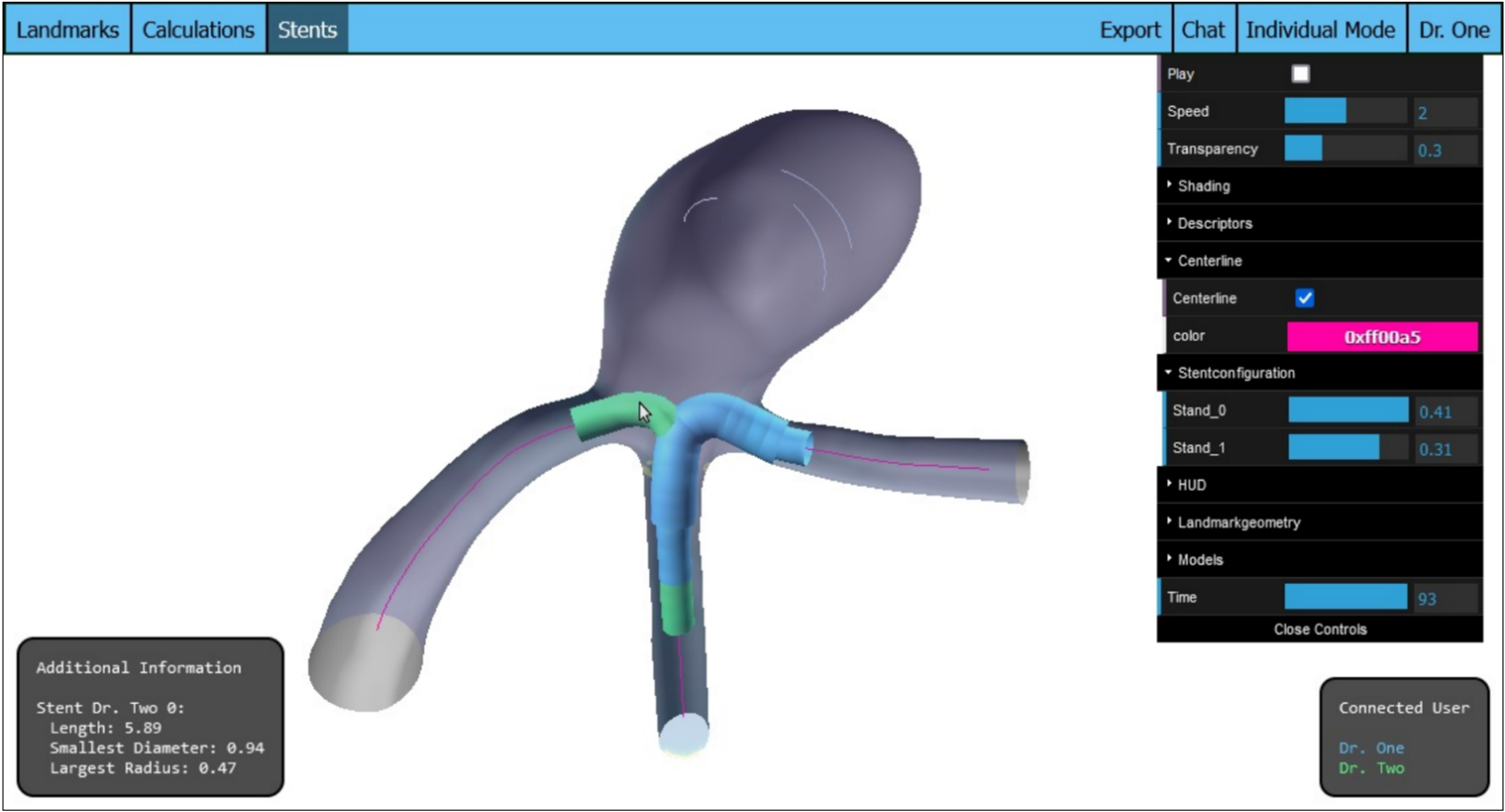
Three stents from multiple users, each with a distinct diameter configuration. The data pertaining to the hovered stent is presented in the information box

The stent configuration feature (R2) requires the addition of new elements to the controls menu. For each stent placed, a slider will appear in the controls, allowing the user to adjust the stent’s diameter. When the slider’s value changes, a new tube geometry representing the stent is generated with the updated diameter. This new geometry replaces the previous configuration, adjusting the stent to reflect the user’s modifications, as shown in Fig. 3.

### Integrated communication tools

In order to facilitate communication over distance and to enhance the user experience for requirement R6, the application was enhanced with the integration of video and text chat functionality. Consequently, a button has been incorporated into the header that enables the display or concealment of a newly created div element, which encompasses all text messages sent by users. Additionally, the chat window incorporates an input field for users to input new messages and a button to initiate a video call.

**Fig. 4:**
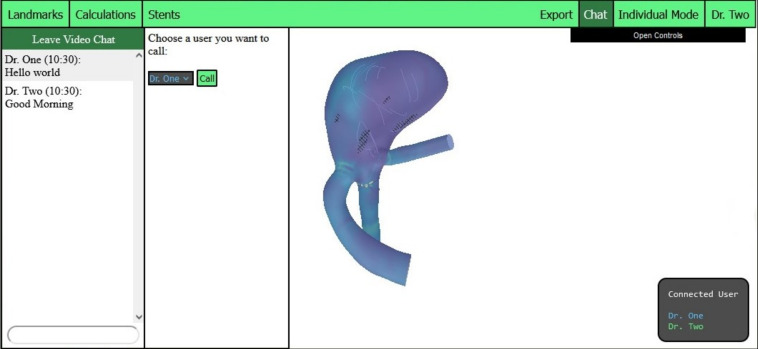
The displayed text chat and selection menu for the video call

Upon clicking the *Start Video Call* button, a window is opened, and a drop-down selection menu is displayed. The selection menu contains a list of all logged-in users. Once a user has been selected from the list and the call button has been clicked, a connection to the selected user is established by the *PeerJS* library. The user selection menu then disappears from the video chat window, and the videos of the call participants become visible. To enhance the usability of the video call, several pop-ups inform the user if someone is calling, if the called user is unavailable, or if a call has ended. The displayed text chat, as well as the selection menu for the video chat, is shown in Fig. 4.

### Work modes

**Fig. 5:**
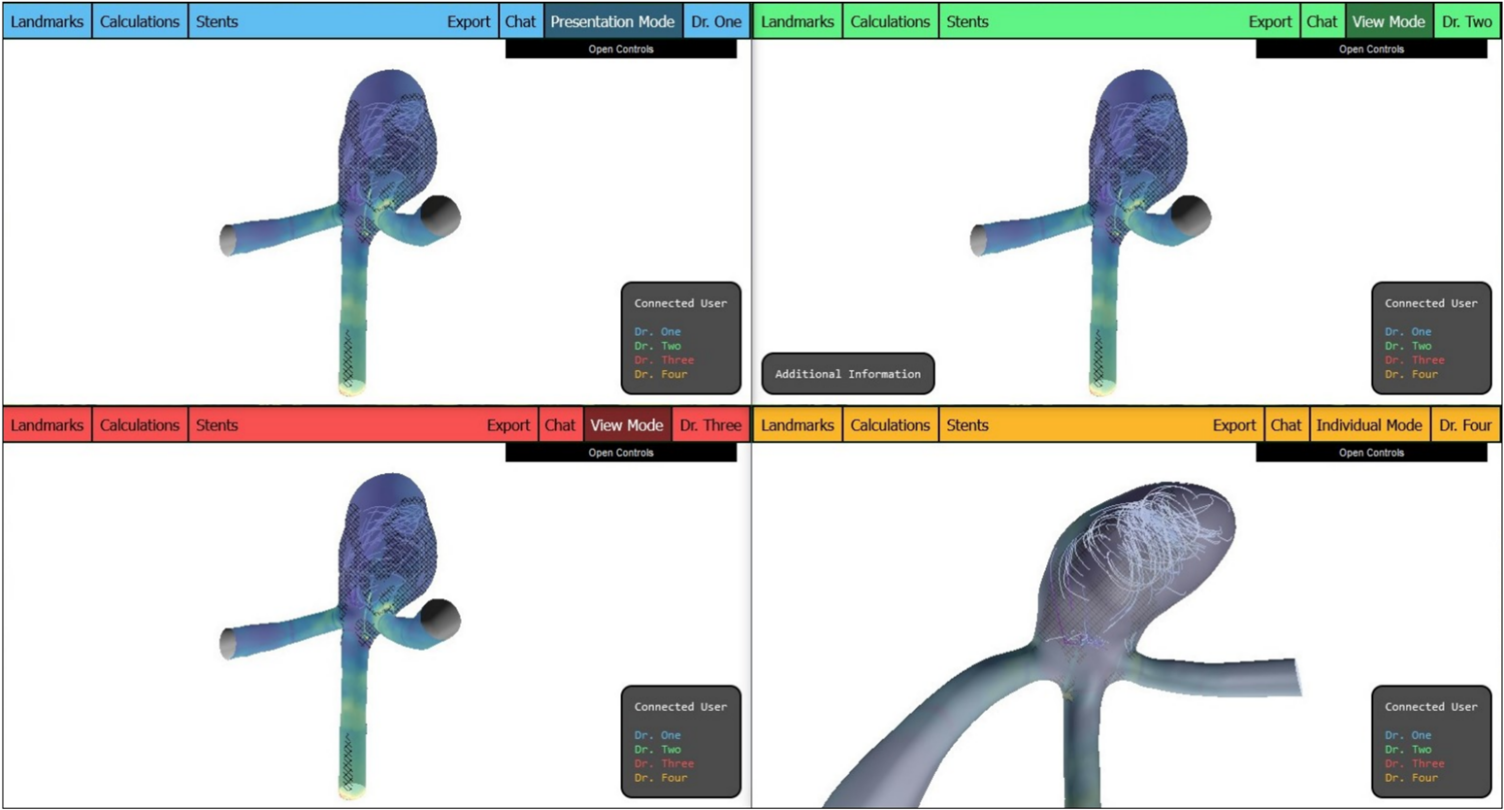
The user with the blue user color who is currently in presentation mode disseminates their personal perspective to all other participants in view mode (green and red colored users). The orange-colored user, operating in individual mode, continues to maintain their own view

The fulfillment of requirement R7 is achieved through the utilization of *the Presentation Mode* and *View Mode*, as illustrated in Fig. 5, in conjunction with the default *Individual Mode*. A button located on the header provides the option of switching between the various modes. Moreover, users are alerted via a pop-up notification when another user changes to presenter mode. Subsequently, the pop-up presents the user with the option to indicate whether they wish to change to view mode. To return to individual mode, the user is required to click the header button a second time.

### Export

The generation of an export document can be initiated by clicking the button labeled *Export* in the header. In this case, a text file will be generated, containing a detailed record of all modifications made during the session. It is necessary to transfer the changes made by each user to the server so that they can be disseminated to all other clients. Upon the implementation of a modification, the server records a concise string, like *‘Dr. One shares the view in presentation mode’*, comprising the completed alteration within an array. In the event that a user requests an export file, the array will be iterated, and all of its constituent strings will be written to the export file.

### Model selection

**Fig. 6:**
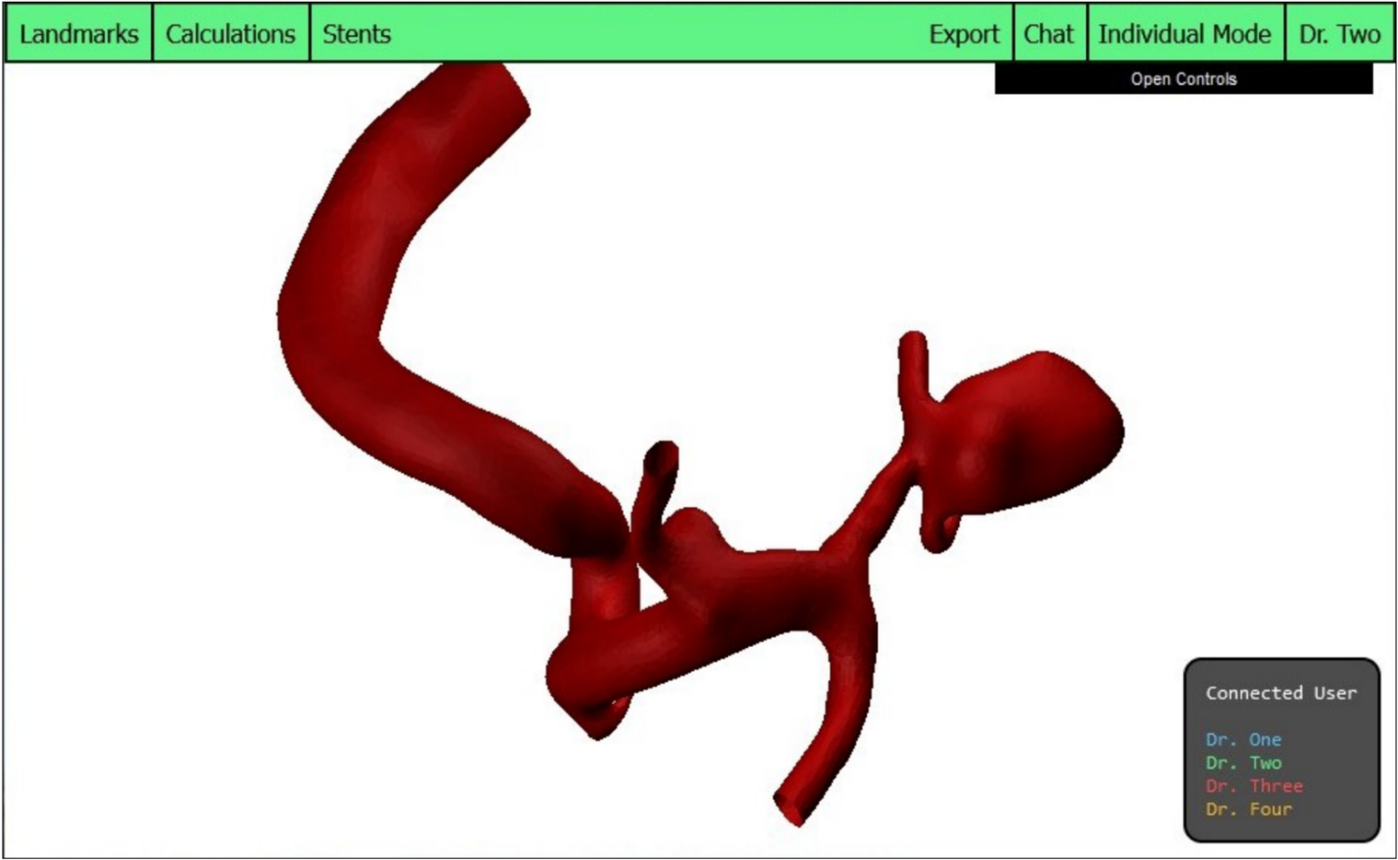
The user interface presents an alternative model for display

To provide physicians with the ability to select and view different datasets (addressing R3), the control menu is further expanded. A drop-down element is incorporated, wherein four distinct models and datasets are presently selectable for exploration. The novel model will be displayed, and the user may then commence exploration. When a model is changed, all modifications will be stored and reloaded automatically, thus enabling physicians to transition between models without losing progress. One of the alternative models from the selection is depicted in Fig. 6.

**Fig. 7:**
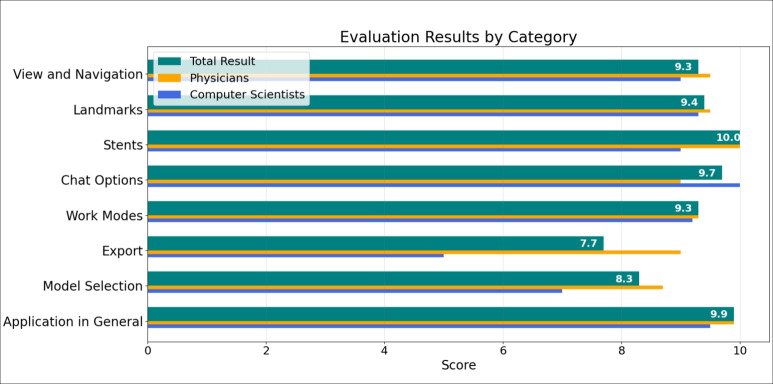
The mean value for each evaluated component of WebCoAneulysis. The results for all participants are shown, as well as the separate results for physicians and computer scientists

## Evaluation

To assess the efficacy of WebCoAneulysis, an extended demonstration was conducted with five participants. Three physicians and two computer scientists with expertise in visualization were asked to use the application alone and together, including all of its functionalities, and then provide feedback. The participants ranged from 26 to 69 years and had varying levels of experience with visualization technologies. Among the physicians, this level ranges from no experience through basic knowledge of Blender and Unreal Engine up to practiced handling of controls within digital scenes in computer games.

The evaluation is structured into eight sections. Seven of these focus on individual features of WebCoAneulysis, while the final section deals with a comprehensive overview of the application. Each section includes a series of tasks designed to evaluate the application’s functionality. Participants are asked to rate the features based on provided statements, using an 11-point Likert Scale, where 10 represents strong agreement or satisfaction and 0 represents the opposite. Additionally, an optional free-text field is available for participants to provide further comments or feedback.

The provided scores were evaluated in accordance with the accompanying statements and categories, as well as by the participants’ professions (computer science and medicine). First, the median score for each statement was determined based on the participants’ responses. Subsequently, the medians of each evaluation section were aggregated to derive the mean score for each feature. Furthermore, the mean score of the physicians and computer scientists was calculated and displayed separately to facilitate a comparison of the scores of both groups. The results are presented in Fig. 7. WebCoAneulysis achieved high point totals in all categories, indicating a high level of satisfaction with the software. The physicians provided higher ratings than the computer scientists across all categories. The subsequent section provides a detailed discussion of the results.

Initially, the participants are required to assess the general overview and navigation of WebCoAneulysis in terms of clarity, comprehensibility, and interaction options. All participants expressed satisfaction with these aspects, particularly the clarity of the application, which was rated on average with 9.3 points.

The evaluation of the landmark feature involved tasks such as adding landmarks of various shapes, annotating and commenting on them, and subsequently removing them. Participants were asked to assess how well this feature supports discussions and surgical planning. On average, the feature received a score of 9.4 points, reflecting strong perceived utility. However, the annotation process requires further improvement. Specifically, the hitbox for the torus geometries is notably small, making it difficult to click and annotate them.

The functionality of the stent was assessed through tasks such as adding and removing the stent within the model, as well as configuring an inserted stent. This feature was rated as the most effective among all functions, achieving a perfect score of 10. Participants highly appreciated its clarity and usefulness in facilitating treatment preparation and discussions.

**Fig. 8:**
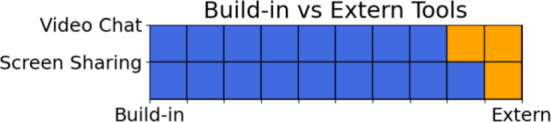
Would you rather use this built-in feature or an external application?

The two chat features were familiar to the participants, as all had prior experience using applications like Zoom or Skype. Consequently, they encountered no difficulties in composing text messages or initiating video calls. On average, the chat functionality received a score of 9.7 points, with participants particularly appreciating the integrated version, as illustrated in Fig. 8. Although participants were more familiar with external chat applications, the integrated chat option was widely regarded as a valuable and supportive feature, especially by computer scientists (see Fig. 7).

**Fig. 9:**
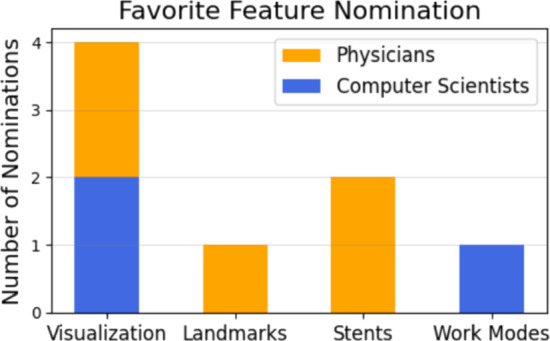
Which feature do you like the most?

The screen-sharing functionality, accessible via the different work modes, was also considered a beneficial feature. To evaluate the effectiveness of the different work modes, participants were instructed to switch to presentation mode and observe their modifications in a secondary tab designated for view mode. The work modes received an average score of 9.3 points, reflecting overall satisfaction with the concept. Participants suggested an additional feature: allowing the presenter to assign a co-presenter with the ability to interact with the scene. Furthermore, as shown in Fig. 8, participants expressed a clear preference for integrating screen-sharing functionality directly into the application, rather than relying on external tools. This preference mirrors their sentiment regarding the chat feature.

The export feature received the lowest score among all features, with an average of 7.7 points. Participants were tasked with exporting and reviewing the generated text file. While physicians expressed high satisfaction with the feature’s utility for documentation and its ease of use, they also noted shortcomings in the clarity and traceability of the exported files. To improve comprehensibility, the structure and format of the information in the exported files need to be revised. Nonetheless, medical professionals hypothesize that this feature would enhance the efficacy and ease of daily work, thereby exerting a profoundly positive influence on clinical routines.

To evaluate the selected models, participants were tasked with selecting an alternative model, making modifications, and reloading the initial dataset. They then assessed whether the features supported their desired workflow for reviewing related cases and ensuring comparability across different datasets. The functionality received an average rating of 8.3 points, reflecting a satisfactory level of acceptance. However, participants suggested that comparability could be further enhanced by incorporating a split-screen visualization, allowing both models to be displayed simultaneously.

In the final section, participants were invited to provide an overall evaluation of the application. They were asked to assess its simplicity, utility in supporting their work, effectiveness in facilitating discussions with colleagues and patients, potential for integration into routine practices, and overall value in treatment preparation. WebCoAneulysis received an overall score of 9.9, reflecting exceptionally high levels of satisfaction. Participants were also asked to identify their favorite feature(s) of WebCoAneulysis. As shown in Fig. 9, the combined visualization was the most frequently highlighted, favored by two physicians and two computer scientists. This was followed by the stent feature, which was particularly appreciated by the physicians.

*‘The aesthetics of this application is nice. The view is very clear, not overloaded and despite the high complexity the application is easy to use.’*, wrote one of the physicians as a free-text answer. Overall, WebCoAneulysis is highly valued for collaborative analysis and discussion support. The stent feature and the visualization itself are particularly noteworthy, and it is stated that the tool would have a positive impact on the treatment planning. Another physician remarked: *‘I would like to use it. The application should be expanded for use in other medical departments and different contexts, such as patient education.’* WebCoAneulysis has the potential to be a supportive tool for all physicians, and its use in patient education is indeed mentioned by several physicians. In particular, they suggest that the visualization could facilitate patient education by presenting patient records in a more accessible and understandable format than traditional medical images of angiograms.

## Conclusion

In conclusion, WebCoAneulysis is specifically designed for collaborative use by medical professionals. The defined tasks and objectives were successfully achieved, resulting in the development of an innovative collaborative application that allows physicians to jointly analyze aneurysm datasets and plan treatments. The evaluation highlighted the effectiveness of WebCoAneulysis, with participants expressing high levels of satisfaction, albeit with a few minor reservations.

Further customization could expand the program’s applicability across a wider range of medical specialties and purposes, including patient education. Such enhancements would further strengthen its utility in supporting physicians in their work.

***Future work***

Despite the positive feedback, there is room for improvement. Incorporating additional analytical tools or alternative visualization techniques from previous Aneulysis versions, such as virtual DSA shading [17], could be a valuable enhancement. Moreover, the detection and analysis of aneurysms can be further optimized. For instance, the automatic marking of aneurysms through detection and segmentation, as demonstrated by Lawonn et al. [21], allows for the concise presentation of their parameters in summary form. This is especially advantageous for datasets comprising multiple aneurysms. The incorporation of an import feature, specifically the capacity to reload and display exported data, would also be a logical and highly beneficial improvement. In addition, the feedback from the evaluation should be carefully considered. Participants proposed incorporating a split-screen functionality for model comparison and enhancing the clarity and structure of the exported text files. It is important to note that the evaluation group is limited in size, with a total of five participants thus far. The objective of the present study was to ascertain whether the application under review was well received. However, the findings must be subjected to further expansion in the future to achieve a more comprehensive understanding of physicians’ needs and to assess the clinical impact. Furthermore, if the application is intended for utilization in routine clinical practice and the execution of extensive tests with non-anonymized data, it is imperative to assess the compliance of the GDPR requirements for the tool.

## Supplementary Information

Below is the link to the electronic supplementary material.

Supplementary file 1 (mp4 183923 KB)
